# Geographic variation in COVID-19 hospital admissions in France: a population-based study: Gregoire Mercier

**DOI:** 10.1093/eurpub/ckac129.689

**Published:** 2022-10-25

**Authors:** G Mercier, V Georgescu, C Baehr, C Riedel

**Affiliations:** 1 Health Data Science Unit, Montpellier University Hospital, Montpellier, France; 2 UMR IDESP, Montpellier University, Montpellier, France; 3 CNRM UMR 3589, Meteo France, Toulouse, France; 4 Internal Medicine Department, Montpellier University Hospital, Montpellier, France

## Abstract

The impact of various environmental, socio-economic, and epidemiological factors on COVID-19 transmission and severity is well-known. However, there is little evidence about the respective role of these factors at the population-level at a national scale. The objective was to identify the environmental and contextual factors that influenced the spread and the severity of COVID-19 at the French department level during the first national lockdown. We performed a national, population-based, retrospective analysis. The cumulative rate of patients admitted for COVID-19 to any public or private acute care hospital from March 31st, 2020 to May 25, 2020 was modelled at the ‘departement’ (hereafter county) level. We used spatial regression models to quantify the aggregated effect of population health status, air pollution, meteorological, and socioeconomic factors. 57,356 patients were admitted to an acute care facility for COVID-19 over the period of interest. At the county level, the age and sex-standardized rate of admission ranged from 0.07 to 3.24 admissions per 1,000 people. After adjustment on the pre-lockdown COVID-19 hospital admission rate, the standardized cumulative rate hospital admission for COVID-19 during the period of interest was significantly and positively associated with the prevalence of diabetes, with the prevalence of mental conditions, and with high cumulative exposure to atmospheric ozone values. It was significantly and negatively associated with high cumulative exposure to ultraviolet radiation. These results suggest that several population-based epidemiological and meteorological factors could have played a role in COVID-19 spread in France. They provide potentially useful insights to design and implement geographically differentiated public health policies.

